# Comparative Analysis of Classifiers for Developing an Adaptive Computer-Assisted EEG Analysis System for Diagnosing Epilepsy

**DOI:** 10.1155/2015/638036

**Published:** 2015-03-05

**Authors:** Malik Anas Ahmad, Yasar Ayaz, Mohsin Jamil, Syed Omer Gillani, Muhammad Babar Rasheed, Muhammad Imran, Nadeem Ahmed Khan, Waqas Majeed, Nadeem Javaid

**Affiliations:** ^1^SMME, National University of Sciences & Technology, Islamabad 44000, Pakistan; ^2^Department of Electrical Engineering, COMSATS Institute of IT, Islamabad 44000, Pakistan; ^3^King Saud University, P.O. Box 92144, Riyadh 11543, Saudi Arabia; ^4^Lahore University of Management Sciences, Lahore 54000, Pakistan; ^5^Department of Computer Science, COMSATS Institute of IT, Islamabad 44000, Pakistan

## Abstract

Computer-assisted analysis of electroencephalogram (EEG) has a tremendous potential to assist clinicians during the diagnosis of epilepsy. These systems are trained to classify the EEG based on the ground truth provided by the neurologists. So, there should be a mechanism in these systems, using which a system's incorrect markings can be mentioned and the system should improve its classification by learning from them. We have developed a simple mechanism for neurologists to improve classification rate while encountering any false classification. This system is based on taking discrete wavelet transform (DWT) of the signals epochs which are then reduced using principal component analysis, and then they are fed into a classifier. After discussing our approach, we have shown the classification performance of three types of classifiers: support vector machine (SVM), quadratic discriminant analysis, and artificial neural network. We found SVM to be the best working classifier. Our work exhibits the importance and viability of a self-improving and user adapting computer-assisted EEG analysis system for diagnosing epilepsy which processes each channel exclusive to each other, along with the performance comparison of different machine learning techniques in the suggested system.

## 1. Introduction

Epilepsy is a chronic neurological disease. The hallmark of this disease is recurring seizures. It has been cited that one out of hundred people suffers from this disorder [1]. Electroencephalography is the most widely used technique for diagnosis of epilepsy. EEG signal is the representation of voltage fluctuations which are caused by the flow of neurons ionic current. Billions of neurons maintain brains electric charge. Membrane transport proteins pump ions across their membranes. Neurons are electrically charged by these membranes. Due to volume conduction, wave of ions reaches the electrodes on the scalp that pushes and pulls the electron on the electrode metal. The voltage difference due to pull and push of the electrons is measured by voltmeter whose readings are displayed as the EEG potential. Neuron generates too small of a charge to be measured by an EEG, and it is the summation of synchronous activity of thousands of neurons that have similar spatial orientation which is measured by an EEG. Unique patterns are generated in the EEG during an epileptic seizure. These unique patterns help the clinicians during diagnosis and treatment of this neurological disorder. That is why EEG is widely used to detect and locate the epileptic seizure and zone. Localization of the abnormal epileptic brain activity is very significant for diagnosis of epileptic disorder.

Usually the duration of a typical EEG varies from few minutes to few hours but in case of prolonged EEG it can even last as long as 72 hours. This generates an immense amount of data to be inspected by the clinician which could prove to be a daunting task.

Advancement in signal processing and machine learning techniques is making it possible to automatically analyse EEG data to detect epochs with epileptic patterns. A system based on these techniques can aid a neurologist by highlighting the epileptic patterns in the EEG up to a significant level. Of course, the task of diagnosis should be left to the neurologist. However, the task of the neurologist becomes efficient as it reduces the data to be analysed and lessens up the fatigue. Along with classification these analysis software programs can also provide simultaneous visualization of multiple channels which helps the clinician in differentiating between generalized epilepsy and focal epilepsy.

It is well known that an epileptic seizure brings changes in certain frequency bands. That is why usually the spectral content of the EEG is used for diagnosis [2]. These are identified as *δ* (0.4–4 Hz), *θ* (4–8 Hz), *α* (8–12 Hz), and *β* (12–30 Hz). Noachtar and Rémi mention almost ten types of epileptic patterns. However, most of the existing work only focuses on one of the epileptic patterns, that is, 3 Hz spike and wave which is a trademark for absence seizure. Other types of the patterns are rarely addressed [3].

Computer-assisted EEG classification involves several stages including feature extraction, feature reduction, and feature classification. Wavelet transform has become the most popular feature extraction technique for EEG analysis due to its capability to capture transient features, as well as information about time-frequency dynamics of the signal [4]. Other previously used feature extraction approaches for epilepsy diagnosis include empirical mode decomposition (EMD), multilevel Fourier transform (FT), and orthogonal matching pursuit [5–9]. Feature extraction is followed by feature reduction to reduce computational complexity and avoid curse of dimensionality. Most commonly, the reduced feature vector consists of statistical summary measures (such as mean, energy, standard deviation, kurtosis, and entropy) of different sets of original (unreduced) features, although other methods such as principal component analysis, discriminant analysis, and independent component analysis have also been used for feature reduction [4, 7, 10, 11]. Feature extraction/reduction is followed by classification using a machine learning algorithm, such as artificial neural networks (ANN), support vector machines (SVM), hidden Markov models, and quadratic discriminant analysis [8, 11–14].

A very important and novel phase of our system is user adaptation mechanism or retraining mechanism. There are multiple reasons according to which introduction of this phase has lots of advantages. During this phase, system will try to adapt its classification as per users desire. It has been cited that sometimes even the expert neurologists have some disagreement over a certain observation of an EEG data. There is also a threat of overfitting by the classifier. In order to keep the classifier improving its performance with the encounter of more and more examples, we have introduced this user adaptive mechanism in our system. We consider the existing systems as dead because they cannot improve their classification rate after initial training. They do not have any mechanism of learning or improvement from neurologists corrective marking [15–17]. The agreement between different EEG readers is low to moderate; our adaption mechanism helps the user in catering this issue as our system tries to adapt the detection according to the users corrective marking. The new corrective markings generate new examples with improved labels. Hence, it populates the training examples with newly labelled ones. So after retraining machine learning algorithms in the system, users adapt to set of choices.

In the next section we will explain our proposed method which will be followed by the results. In the results section, we will explain how SVM performs better than QDA and ANN in our proposed method. We will also show that exclusive processing of each channel results in a significant improvement in the classification rate. Here “epileptic pattern” and “epileptic spikes” will be used as an alternative to each other.

## 2. Proposed Method

Computer-aided EEG analysis systems use the neurologists marking and labelling of the EEG data as a benchmark to train themselves during initial training phase. But after initial training phase, these systems have no simple mechanism for these neurologists to improve systems classification after encountering any false classification. So we have proposed a method by which systems classification can be improved by the user in a relatively simpler way. This analysis system only tries to detect the epileptic spikes as mentioned by Noachtar and Rémi. Later it adapts its detection of epileptic spikes exclusively for every user (Figure 4).

In this proposed system, we are processing each channel for each epileptic pattern exclusive to each other. This exclusive processing of each channel not only helps the user in diagnosing localized epilepsy but also eases up the classifiers job. We have considered that different epileptic patterns are independent to each other and their separate handling will help us in avoiding error propagation from one epileptic pattern type detection to the other. Our systems working has two major phases (A) initial training phase and (B) adaptation phase. These two major phases have further three parts which are (1) feature extraction, (2) feature reduction, and (3) classification. Next we will briefly explain all of these steps.

### 2.1. Initial Training

#### 2.1.1. Feature Extraction

To decide which parts of the signal are epileptic and which are not we first divided whole of the signal in small chunks known as epochs. Then DWT was applied on those epochs so that visibility of epileptic activity can be enhanced which is distinguished by some spectral characteristics. These features are then processed to make them more suitable for the classification technique.

**Figure 1:**
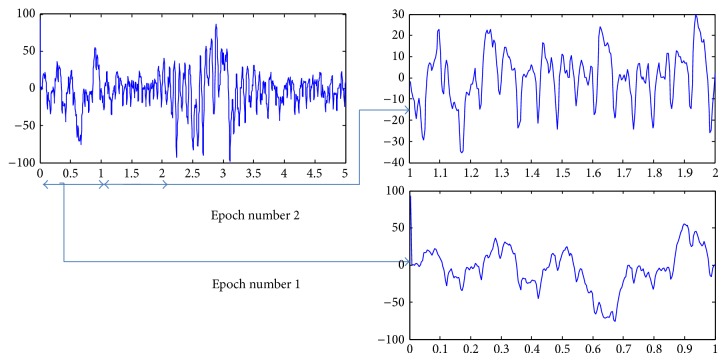
Epoch size is 1 sec.

*(a) Epoch Size.* The first important part of the feature extraction is epoch selection. Epoch is a small chunk of the signal which is processed at a time. The size of the epoch is very important. The larger it is the less accurate it will be. The smaller it is the higher the processing time will be. After testing different epoch sizes, we found epoch size of nonoverlapping 1 sec window to be best yielding in terms of accuracy. It also reestablished the work of Seng et al. [18] (Figure 1).

*(b) DWT*. As discussed in Introduction, spectral analysis is very informative while examining the epilepsy suspected patients EEG. There are profound advantages of wavelet decomposition which is a multiresolution analysis technique. A multiresolution analysis technique allows us to analyse a signal for multiple frequency resolutions while maintaining time resolution unlike a normal frequency transform. Wavelet decomposition allows us to increase frequency resolution in the spectral band of our interest while maintaining the time resolution; in short we can decimate these values simultaneously in time and frequency domain.

During wavelet transform, the original epoch is split into different subbands: the lower frequency information is called approximate coefficients and the higher frequency information is called detailed coefficients. The frequency subdivision in these subbands helps us in analysing different frequency ranges of an EEG epoch while maintaining its time resolution [4, 8, 13]. The choice of coefficients level is very important as the epileptic activity only resides in the range of 0–30 Hz. Coefficients levels of the DWT are determined with respect to sampling frequency. So, the detailed levels of interest are adjusted on the run according to the sampling frequency such that we may get at least one exact value of the closest separate *δ* (0.4–4 Hz), *θ* (4–8 Hz), *α* (8–12 Hz), and *β* (12–30 Hz) components of the signal. We discarded all the detailed coefficient levels which were beyond the 0–30 Hz range.

Then DWT was applied on each epoch with Daubechies-4 (db4) as mother wavelet. The detailed coefficient levels of the DWT were determined with respect to sampling frequency.

*(c) Statistical Features*. After the selection of detailed coefficients which represent the frequency band of our interest, we calculated the statistical features by calculating the mean, standard deviation, and power of these selected wavelet coefficients. These statistical features are inspired from Subasi and Gursoy work [13].

*(d) Standardization*. These statistical features were then standardized. During training stage *z*-score standardization was applied on these features [19]. This standardization is just like usual *z*-score normalization, but as we do not know the exact mean and standard deviation of the data (to be classified) during classification/test stage, we used the mean and standard deviation of the training examples during training stage for standardizing (normalizing) the features during classification stage. We normalized the features by subtracting and dividing them by training examples mean and standard deviation, respectively.

### 2.2. Feature Reduction

In order to avoid overinterpretation by redundant data and misinterpretation by noisy data we applied feature reduction method. Inclusion of this part increases the processing time, thus exacerbating the latency.

Dimensionality reduction using principal component analysis (PCA) is based on a very important trait that is variance of the data. PCA develops the nonlinear mapping in such a way that it maximizes the variance of the data, which helps us in discarding that part of the data which is marked by lesser variances. This reshaping and omission not only removes the redundant data but also lessens up the noise.

During training stage PCA was applied on these features in order to reduce the redundant and/or noisy data. We kept the components which projected the approximate 95% of the total variance. We were able to reduce the 21 features into 9. Then we fed these reduced features to classifiers trainer. Here as per our observation we again assumed that the EEG data is stationary for a small length. So, during the testing stage, we took the PCA coefficients matrix from training stage and multiplied it with the standardized statistical features of the blind test data and then fed the top 9 features to classifier.

### 2.3. Classification

Classification is a machine learning technique in which new observations belonging to a category are identified. This identification is based on the training set which contains the observations with known labelling of their category. These observations are also termed as features. We tried three types of classification methods: (1) SVM, (2) QDA, and (3) ANN (Figure 3).

The reduced features were fed to these classifiers. Here the reduced features mean that those statistical features of the selected wavelet coefficients are reduced using PCA as described in previous section. All of the three processing parts were exclusive for each channel and each epileptic pattern. So like previous parts the classifiers were also trained and tested exclusively for each channel.

Our system requires individual labelling of channels. There is a separate classifier for each channel and for each epileptic pattern type. So, the total number of classifiers is equal to the product of total number of channels by ten where ten represents the number of epileptic pattern described by Noachtar and Rémi [3].

*(e) Support Vector Machine*. Support vector machine (SVM) is a supervised learning models machine learning technique. SVM tries to represent the examples as points in space which are mapped in a way that points of different categories can be divided by a clear gap that is as wide as possible. Afterwards, that division is used to categorise the new test examples based on which side they fall on.

*(f) Quadratic Discriminant Analysis*. Quadratic discriminant analysis (QDA) is a widely used machine learning method among statistics, pattern recognition, and signal processing to find a quadratic combination of features which are responsible for characterizing an example into two or more categories. QDAs combination of discriminating quadratic multiplication factors is used for both classification and dimensionality reduction.

*(g) Artificial Neural Network*. Artificial neural network (ANN) is a computational model which is inspired from animals central nervous system. That is why ANN is represented by a system of interconnected neurons which are capable of computing values as per their inputs. In ANN training, the weights associated with the neurons are iteratively adjusted according to the inputs and the difference between the outputs with expected outputs. The iteration gets stopped when either the combination of neurons starts generating the expected results within an error of a tolerable error range or the iteration limit finishes up.

### 2.4. Adaption Phase (Retraining/User Adaptation Mechanism)

In order to keep the classifier improving its performance with the encounter of more and more examples, we have introduced a user adaptive mechanism in our system. Our system allows the user to interactively select epochs of his choice by simply clicking on the correction button. While using our system, when a user thinks that a certain epoch is falsely labelled/categorised, our system allows him to interactively mark mark that label as a mistake. These details will be saved in a log in the background and they will be used to retrain the classifier to improve its classification rate and adapt itself according to the user with the passage of time. When the user is going to select the retraining option in our system, then classifiers will retrain themselves on the previous and the newly logged training examples. As every user has to log in with his personal ID, every corrective marking detail will only be saved in that user's folder and only classifier will update itself for that user. Hence, the systems classifier tries to adapt itself according to that user without damaging anyone else classification.

The concept behind the inclusion of the retraining is that if there is more than one example with same attributes but different labels, the classifier is going to get trained to the one with most population. The user's corrective marking will increase the examples of his choice, thus making that classifier adapt itself to the user's choice in a trivial way. Every user will have exclusive classifiers trained for him and his marking will not affect other users' classifier. As we know, the users sometimes do not agree on the choice of the epileptic pattern or its type. The exclusive processing for each user will help the same software keep the system trained for every user and it will also let different users compare their markings with each other.

We do not have any standard right now to measure which neurologist is the most righteous among a disagreeing group of neurologist users. So we kept the corrective markings of each user to his account so that it may not interfere with the one who may not agree on his choice. So, the developed system is used to facilitate the neurologist's selection to the user according to his own choice and after initial training on every retraining it tries to adopt more users. This system does not want to dictate to the neurologist but rather learn from him to adapt him to save his time.

We want the classifier to think like the user and supplement him by highlighting the epochs of his choice, so the gold standard after few retraining mechanisms will be the user himself. Already tested examples with new labels inclusion in the training examples for the retraining will bias the classifiers choice in favour of user.

## 3. Experimentation

In this section, we will discuss the results in detail. At first, we will describe the datasets which we used to train, test, and validate our method. Then we will discuss their versatility (Figure 7).

### 3.1. Dataset

Two labelled datasets of epilepsy suspected surface EEG data were available to us. Both of these datasets have lots of versatility in between them in terms of ethnicity, age, gender, and equipment. The datasets available to us were about generalised absence seizure which is characterized by the 3 Hz spike and wave epileptic pattern in almost each channel. That is why we have classification results available only for one type of epilepsy which is absence seizure.

#### 3.1.1. CHBMIT

This database is the online available surface EEG dataset [20] which is provided by Children Hospital Boston and Massachusetts Institute of Technology and it is available at physioNet website [10]. It contains 916 hours of 23 channels scalp EEG recording from 24 epilepsy suspected patients. This ECG recording is sampled at 256 Hz with 16-bit resolution. The 23rd channel is same as 15th channel (Table 1).

#### 3.1.2. PIMH

The second database of EEG datasets is provided by our collaborator at Punjab Institute of Mental Health (PIMH), Lahore. Its sampling frequency is 500 Hz and it was recorded on 43 channels (among which 33 channels are for EEG). This dataset consists of 21 patients EEG recording.

### 3.2. Features

#### 3.2.1. Feature Extraction

**Figure 2:**
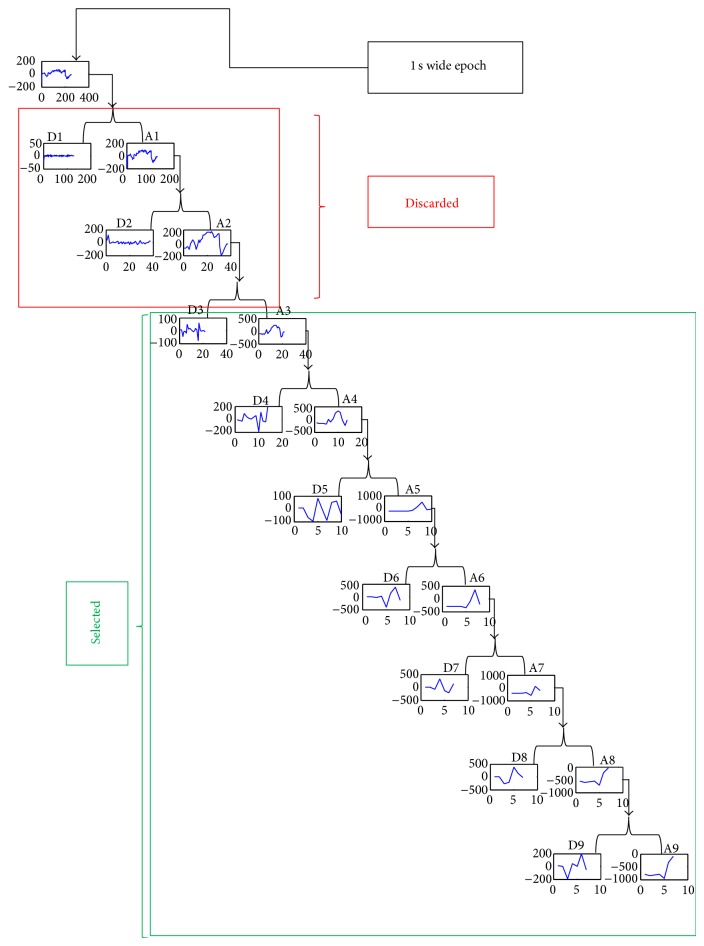
Selection of DWT detailed coefficients for a 256 Hz sampled 1 sec wide epochs EEG signal.

**Figure 3:**
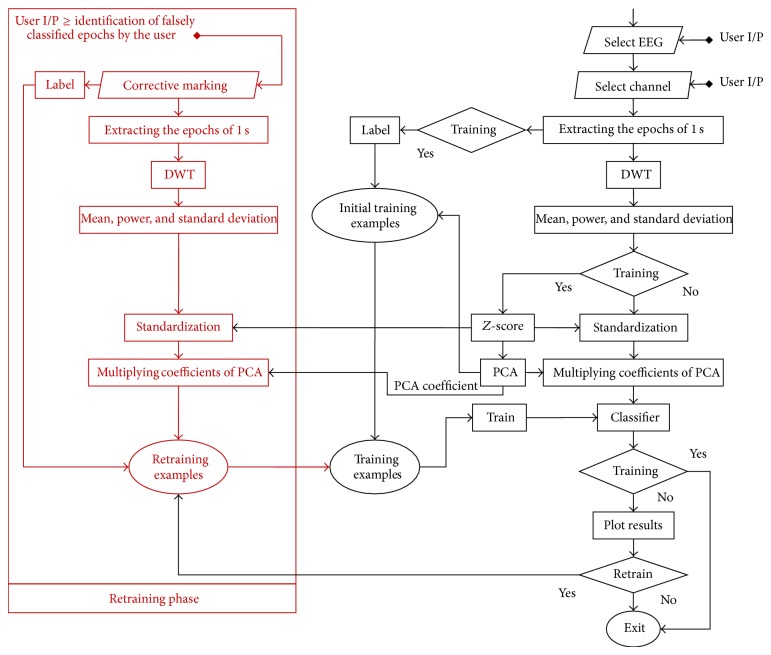
Workflow of a single channel.

**Figure 4:**
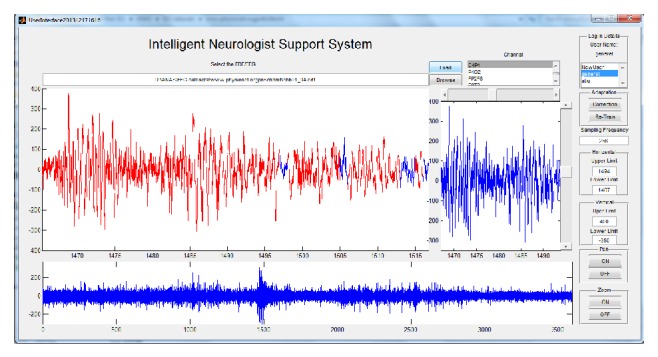
iNSS interface.

Data which interests us lies in between the frequency range of 0.3 Hz to 30 Hz. So after applying DWT with db4 mother wavelet, we have to select detailed coefficients with this frequency range. So in case of 256 Hz sampled CHBMIT dataset, we have to go to at least 3 levels of decomposition and discard the earlier two as it is demonstrated in Figure 2. In order to get the discriminating information between different types of epileptic patterns and identifying them correctly without mistaking them with each other, decomposition of this detailed coefficient further in Beta, Alpha, Theta, and Delta is hugely helpful. So we further decomposed them until the 7th level. Hence, we used the DWTs detailed coefficients of levels 3, 4, 5, 6, and 7 for 256 Hz sampled CHB-MIT dataset (Table 2).

After the selection of the wavelet coefficients, we calculated the statistical feature out of them. The statistical features were the mean, power, and standard deviation of all of the selected coefficients.

In case of 512 Hz sampled PIMH dataset, we used the DWTs detailed coefficients of levels 4, 5, 6, 7, and 8.

After the selection of detailed coefficients, we calculated the statistical feature out of them. The statistical features were the mean, power, and standard deviation of all of the shortlisted detailed coefficients.

#### 3.2.2. Standardization

**Figure 5:**
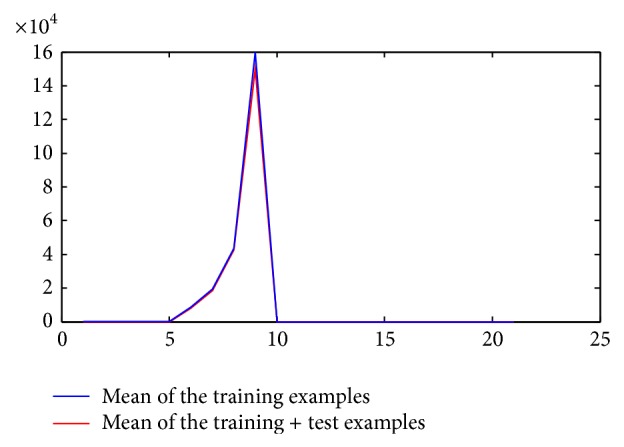
Relationship between channel's number and mean value (test + training examples).

**Figure 6:**
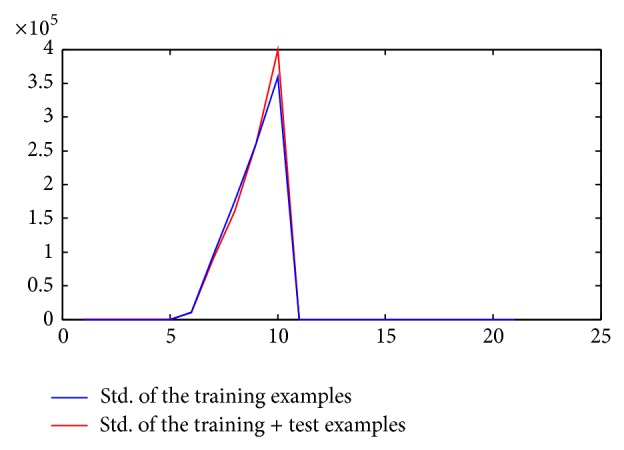
Relationship between channel's number and standard deviation value (test + training examples).

**Figure 7:**
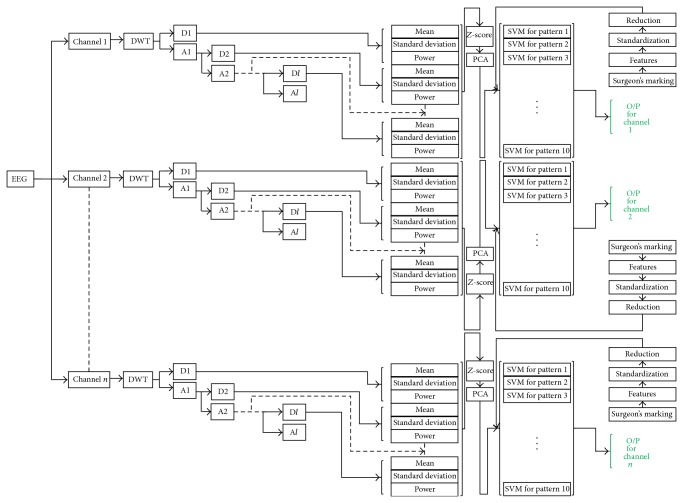
Flowchart.

During training stage, we first used simple *z*-score normalization to standardize the features [19] before applying feature reduction. But the real issue arose when we tried to normalize them during testing stage. One way of doing this is that we keep all of the examples and apply *z*-score on them along with the new test data. Instead of this time taking process, we made an assumption on our observation that mean and standard deviation does not deviate a lot. It is analysed in this study that the EEG time series are assumed to be stationary over a small length of the segments. So we used the mean and standard deviation of the training examples from the training stage to normalize the test examples. Figures 5 and 6 illustrate our observation, in which you can see that there is not much deviation in train and train + test examples mean and standard deviation.

### 3.3. Classifier

Classification is used in machine learning to refer to the problem of identifying a discrete category to which a new observation belongs. Observations with known labels are used to train a classification algorithm or classifier using features associated with the observation. For CHBMIT database, we had to train 220 classifiers in initial training stage. The calculation behind 220 is the 22 channels multiplied by 10 types of epileptic pattern. The 23rd channel was same as 15th channel. For PIMH dataset 330 classifiers were trained where 33 channels of EEG were utilized. We tried three different classifiers and found SVM to be the most accurate.

We have used blind validation mechanism for the ten different feature data distributions to estimate the classification performance. These 10 different and separate blind data distributions were taken from a huge set of EEG dataset. These 10 data distributions we randomly divided into two groups. We trained our classifier on one half of the distribution and tested it on the other half. We repeated that on all ten distributions. Then we calculated the average of the classification rate for the all ten distributions.

3229 out of 3297600 epochs were randomly taken for ten times from CHBMIT dataset. Each time half of them were used to train and half of them were used to test the initial classification. The average of the sensitivity, specificity, and accuracy for these ten distributions is considered as the initial training phase performance.

Same approach was applied on PIMH datasets where 3229 out of 24097 epochs were randomly taken from PIMH dataset for the six times instead of ten times.

Due to unavailability of the non-3 Hz spike and wave epileptic EEG data, currently we have only classification rates for generalized absence seizure.

#### 3.3.1. Exclusive Processing

**Figure 8:**
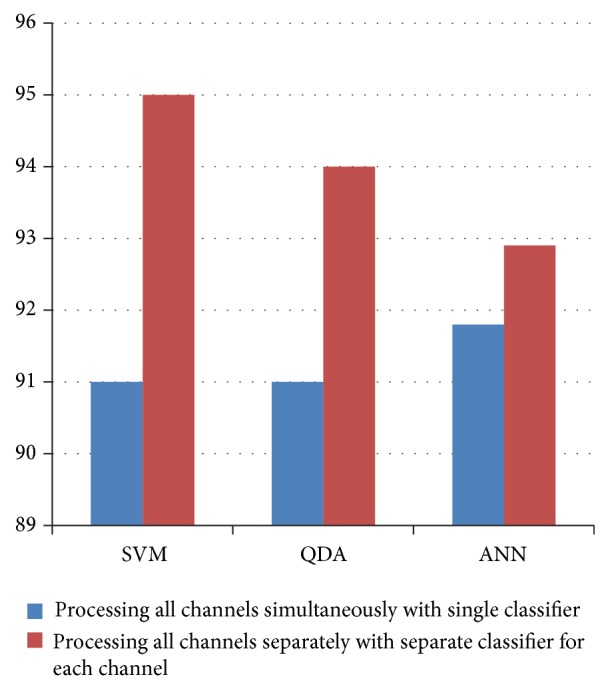
Accuracy relationship of different classifiers and their classification rate.

In this study, we have analysed that even in the case of absence seizure epileptic patterns do not appear in the exact same way in each channel. Handling of each channel exclusive to each other was also another very important decision. We tested the classification in both ways, that is, one classifier for all of the channels at once versus one separate classifier for each channel (Figure 8).

This processing of each channel exclusive to each other improved over average accuracy from approximately 91% to approximately 95% in case of SVM. So for SVM, there is a significant improvement of 4% by this change. In case of QDA accuracy rose from 91% to 94% with an improvement of 3% and in case of ANN it rose from 91.8% to 92.9% with an improvement of 1.1%.

Results show that SVM suites our method in the most efficient way. ANN has a lesser classification time and LDA has a lesser training time as compared to SVM, but considering the sensitivity and classification improvement through corrective marking, we think that SVM is the better choice than LDA and ANN. In upcoming sections, we have shown the results for all three types of classifier.

*(a) Adaptation Mechanism*. To test the adaptation mechanism 807 corrective epochs were marked by the user for a CHBMIT dataset file, and he marked the same amount of epochs for each channel. These corrective markings were saved in his log as training examples. These corrective markings as the new examples along with the 32290 epochs of initial training stage were used to retrain the classifier. The number 32290 has come from the 3229 randomly selected epochs from whole of the CHBMIT dataset for the ten separate times during initial training phase. Then later the performance of the classifier after retraining was judged again on another random 3000 epochs (Figure 14).

In case of PIMH dataset 57 corrective epochs were selected for PIMH dataset. This time 19374 epochs of the PIMH dataset were used along with the 57 corrective markings, as for the PIMH we randomly selected the 3229 numbers of epochs for the six times. The retrained classifier was tested on the 2361 remaining epochs.

*(b) Support Vector Machine*. We used the support vector machine classifier package available in MATLAB Bioinformatics Toolbox. We found linear kernel to be the most accurate SVM kernel with 50 as the box constraint.

*(c) CHBMIT*. For CHBMIT dataset, initial training of the classifier resulted in 96.3% average accuracy, 97.4% average specificity, and 93.5% average sensitivity for 3 Hz spike and wave which is a characteristic of absence seizure. After initial training our specificity is better than that of Shoeb [10] and Nasehi and Pourghassem [21] who used the same dataset to validate their technique with different features and application technique. This shows that our technique is providing better results even at the initial training phase (Figure 11).

In Table 3 we have shown the average initial classification and retrained classification results of our system for each channel. In this system, we have shown that after correction of few epochs there is a visible improvement in the systems classification. The average accuracy of the system rose from 95.5% to 96.3%.

*(d) PIMH*. For PIMH dataset, initial training of the classifier resulted in 90% average accuracy, 94% average specificity, and 80% average sensitivity for 3 Hz spike and wave which is a characteristic of absence seizure (Figure 12).

In Table 4, we have shown the average initial classification and retrained classification results of our system for each channel. Table 4 shows that our technique is robust and it works also on a different dataset. The average accuracy of the system rose from approximately 89% to 90%.

#### 3.3.2. Discriminate Analysis

We used the discriminant analysis package available in MATLAB Statistics Toolbox. We found pseudoquadratic to be the best performing discriminate type with uniform probability.

*(a) CHBMIT*. For CHBMIT dataset, initial training of the classifier resulted in 94% average accuracy, 96% average specificity, and 90% average sensitivity for 3 Hz spike and wave which is a characteristic of absence seizure. After initial training our specificity is better than that of Shoeb [10] and Nasehi and Pourghassem [21] (Figure 13).

In Table 5, we have shown the average initial classification and retrained classification results of our system for each channel. In this system, we have shown that after correction of few epochs there is visible improvement in the systems classification. The average accuracy of the system rose from 94% to 95%.

*(b) PIMH*. For PIMH dataset, initial training of the classifier resulted in 90% average accuracy, 95% average specificity, and 73% average sensitivity for 3 Hz spike and wave which is a characteristic of absence seizure.

In Table 6, we have shown the average initial classification and retrained classification results of our system for each channel. Table 6 shows that our technique is robust and it works also on a different dataset. The average accuracy of the system rose from approximately 89% to 90%.

#### 3.3.3. Artificial Neural Network

We used feedforward backpropagation package available in MATLAB Neural Network Toolbox and found Levenberg-Marquardt to be the best method, with 0.05 learning rate.

**Figure 9:**
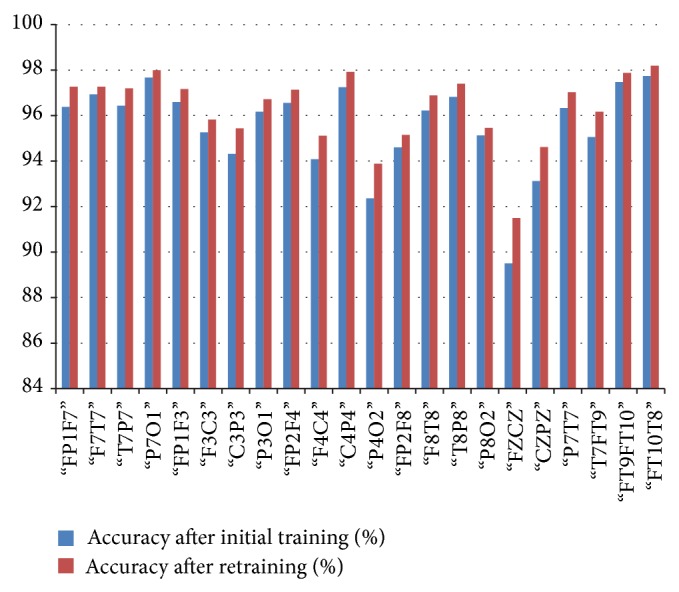
Relation between average classification rate and accuracy of the channel after initial training and retaining.

*(a) CHBMIT*. For CHBMIT dataset, initial training of the classifier resulted in 92.88% average accuracy, 98.66% average specificity, and 75.75% average sensitivity for 3 Hz spike and wave which is a characteristic of absence seizure (Figure 9).

In Table 7, we have shown the average initial classification and retrained classification results of our system for each channel. In this system, we have shown that after correction of few epochs there is visible improvement in the systems classification. The average accuracy of the system rose from 92.88% to 93.96%.

**Figure 10:**
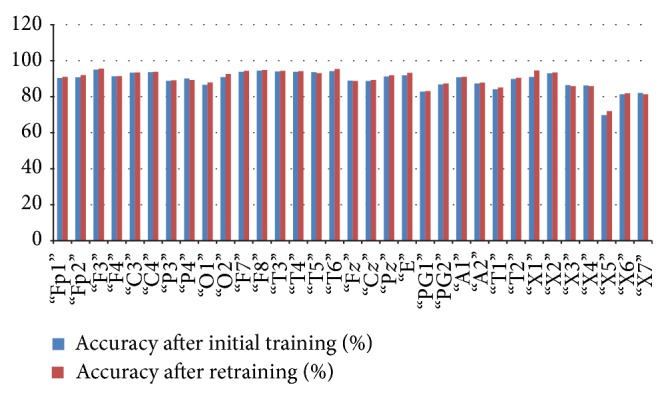
Relation between average classification rate and accuracy of the channel after initial training and retaining.

**Figure 11:**
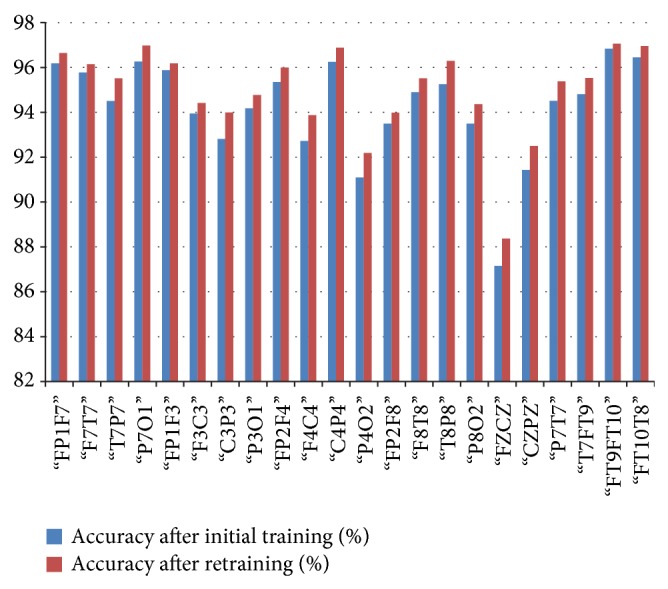
Relation between average classification rate and accuracy of the channel after initial training and retaining.

**Figure 12:**
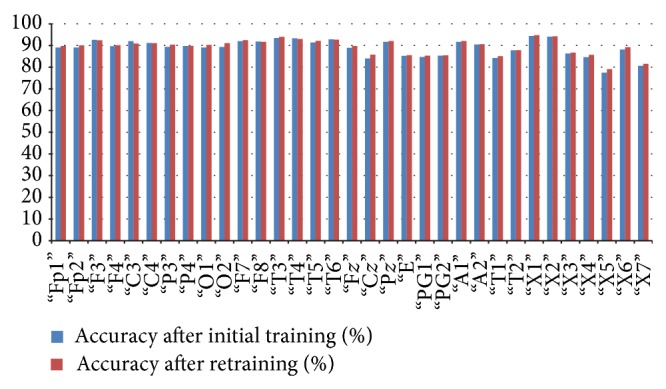
Relation between average classification rate and accuracy of the channel after initial training and retaining.

**Figure 13:**
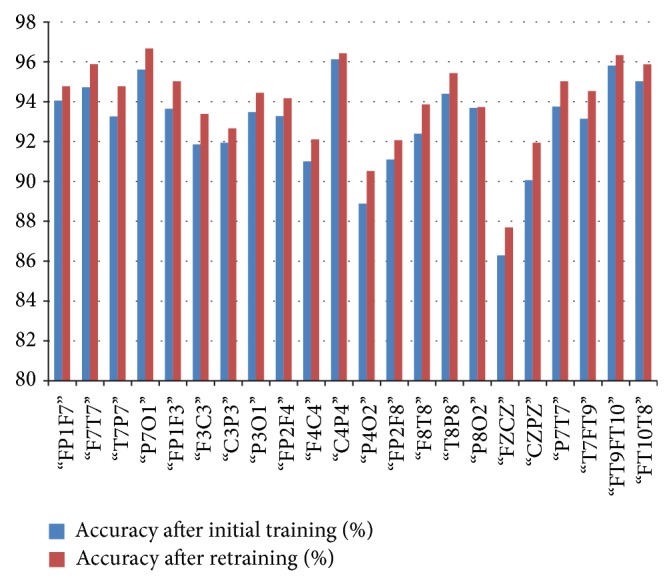
Relation between average classification rate and accuracy of the channel after initial training and retaining.

**Figure 14:**
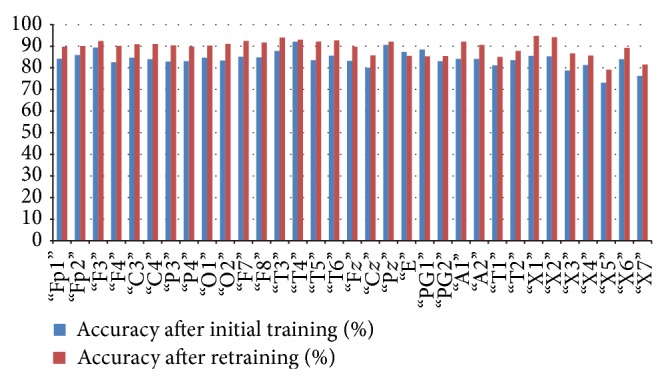
Relation between average classification rate and accuracy of the channel after initial training and retaining.

*(b) PIMH*. For PIMH dataset, initial training of the classifier resulted in 84% average accuracy, 94.8% average specificity, and 56.5% average sensitivity for 3 Hz spike and wave which is a characteristic of absence seizure (Figure 10).

In Table 8, we have shown the average initial classification and retrained classification results of our system for each channel. Table 8 shows that our technique is robust and it works also on a different dataset. The average accuracy of the system rose from approximately 84% to 85.43%.

## 4. Discussion and Future Work

Computer-assisted analysis of EEG has tremendous potential for assisting the clinicians in diagnosis. A very important and novel phase of our system is user adaptation mechanism or retraining mechanism. Introduction of this phase has importance in many aspects. In this phase, system tries to adapt its classification according to users desire. Moreover, this technique personalizes the classifiers classification. It has been cited that sometimes even the expert neurologists have some disagreement over a certain observation of an EEG data. This system will be useful for disagreeing users and it will also help them in comparing their results with each other.

There is also a threat of overfitting by the classifier. In order to keep the classifier improving its performance with the encounter of more and more examples, we have introduced this user adaptive mechanism in our system. We consider the existing systems as dead because these cannot improve their classification rate after initial training (during software development). The self-improving mechanism after deployment makes our tool alive. This system can be made part of the whole epileptic diagnosis process. It will highlight the epileptic spikes among the whole EEG, thus leading to reduced fatigue and time consumption of a user. We obtained high classification accuracy on datasets obtained from two different sites, which indicates reproducibility of our results and robustness of our approach.

In the future, we are planning to make this a web based application; neurologists can log in and consult each other's reviews about a particular subject. This will make our system experience a whole versatility of examples and learn from all of them. Integration of the video and its automatic analysis (video EEG) can help a neurologist in diagnosing epilepsy in a better way, whereas this can also help him in distinguishing between psychogenic and epileptic seizures. We would also be investigating how much overfitting is an issue in the reported performances which are now even touching 100% based on some claims. There is a need for method/criteria which could limit these algorithms improving their detection on a limited number of available examples.

This system is made keeping in mind that we have to facilitate the neurologist by supplementing him in the analysis of the EEG. We do not want to enforce the classification of the EEG data on a user.

In the future, we will also include a slider in the system which will allow the user to adjust the sensitivity and specificity before retraining. This assisting system is more like a detection tool which is continuously learning with encounter of better examples. More and better examples will certainly improve its performance. The agreement between different neurologists over the EEG readings is low to moderate. If we could find the agreement on at least few of the epileptic patterns correspondence with epileptic disease then we can take this tool further ahead and use it for diagnosis instead of just assistance.

One of the biggest limitations to this study is the unavailability of non-3 Hz spike and wave data. Even though we have included the data features of the entire epileptic frequency ranges exclusive to each other, proof testing on the data will certainly prove worthy for the progress of these assisting tools toward a diagnostic tool.

## Figures and Tables

**Table 1:** This table describes the affiliation of detailed coefficients with epileptic frequency band of interest for 256 Hz sampled CHB-MIT dataset.

Epileptic frequency range	Detailed coefficients' level
Beta (*β*)	CD3 (32 Hz to 16 Hz)
Alpha (*α*)	CD4 (16 Hz to 8 Hz)
Theta (*θ*)	CD5 (8 Hz to 4 Hz)
Delta (*δ*)	CD6 (4 Hz to 2 Hz)
Delta (*δ*)	CD7 (2 Hz to 1 Hz)

**Table 2:** This table describes the affiliation of detailed coefficients with epileptic frequency band of interest for 512 Hz sampled PIMH dataset.

Epileptic frequency range	Detailed coefficients' level
Beta (*β*)	CD4 (31.2 Hz to 15.6 Hz)
Alpha (*α*)	CD5 (15.6 Hz to 7.8 Hz)
Theta (*θ*)	CD6 (7.8 Hz to 3.9 Hz)
Delta (*δ*)	CD7 (3.9 Hz to 2 Hz)
Delta (*δ*)	CD8 (2 Hz to 1 Hz)

**Table 3:** First column shows the channel label, the second column shows the initial training accuracy, and third one shows the marked correction by a neurologist and the last one shows the final accuracy.

Channel	Accuracy after initial training (%)	Number of epochs marked by the user	Accuracy after retraining (%)
FP1F7	96.3805	807	97.2696
F7T7	96.9302	807	97.2645
T7P7	96.4295	807	97.1892
P7O1	97.6660	807	97.9844
FP1F3	96.5932	807	97.1636
F3C3	95.2557	807	95.8268
C3P3	94.3129	807	95.4354
P3O1	96.1653	807	96.7156
FP2F4	96.5520	807	97.1408
F4C4	94.0773	807	95.1094
C4P4	97.2442	807	97.9201
P4O2	92.3547	807	93.8824
FP2F8	94.5957	807	95.1480
F8T8	96.2118	807	96.8850
T8P8	96.8148	807	97.3952
P8O2	95.1268	807	95.4580
FZCZ	89.5010	807	91.4936
CZPZ	93.1258	807	94.6167
P7T7	96.3280	807	97.0216
T7FT9	95.0556	807	96.1656
FT9FT10	97.4703	807	97.8714
FT10T8	97.7348	807	98.1905

**Table 4:** First column shows the channel label, the second column shows the initial training accuracy, and third one shows the marked correction by a neurologist and the last one shows the final accuracy.

Channel	Accuracy after initial training (%)	Number of epochs marked by the user	Accuracy after retraining (%)
Fp1	90.3692	57	90.9779
Fp2	90.7681	57	91.9478
F3	95.0380	57	95.6088
F4	91.3297	57	91.3627
C3	93.3561	57	93.3813
C4	93.6448	57	93.8244
P3	88.7797	57	89.0716
P4	90.1133	57	89.3159
O1	86.5848	57	87.8883
O2	90.8300	57	92.5787
F7	93.7438	57	94.3815
F8	94.4563	57	94.8470
T3	93.9221	57	94.3731
T4	93.8322	57	94.1035
T5	93.5670	57	93.1092
T6	94.1287	57	95.3485
FZ	88.9061	57	88.6571
CZ	88.6708	57	89.2548
PZ	91.2450	57	91.8549
E	91.8499	57	93.2420
PG1	82.7552	57	83.0715
PG2	86.7638	57	87.3141
A1	90.7149	57	91.0081
A2	87.3506	57	87.7646
T1	84.1250	57	85.0581
T2	89.7853	57	90.4835
X1	90.9339	57	94.5756
X2	92.9365	57	93.3816
X3	86.3529	57	85.7929
X4	86.2634	57	85.8934
X5	69.7540	57	71.9493
X6	81.2648	57	81.9323
X7	82.0801	57	81.3344

**Table 5:** First column shows the channel label, the second column shows the initial training accuracy, and third one shows the marked correction by a neurologist and the last one shows the final accuracy.

Channel	Accuracy after initial training (%)	Number of epochs marked by the user	Accuracy after retraining (%)
FP1F7	96.1757	807	96.6399
F7T7	95.7686	807	96.1467
T7P7	94.5025	807	95.5098
P7O1	96.2598	807	96.9693
FP1F3	95.8724	807	96.1773
F3C3	93.9484	807	94.4194
C3P3	92.8110	807	93.9872
P3O1	94.1724	807	94.7690
FP2F4	95.3442	807	95.9999
F4C4	92.7255	807	93.8686
C4P4	96.2509	807	96.8740
P4O2	91.0961	807	92.1808
FP2F8	93.4976	807	93.9840
F8T8	94.8939	807	95.5075
T8P8	95.2540	807	96.2940
P8O2	93.4941	807	94.3612
FZCZ	87.1507	807	88.3645
CZPZ	91.4283	807	92.4912
P7T7	94.5118	807	95.3794
T7FT9	94.8031	807	95.5306
FT9FT10	96.8328	807	97.0555
FT10T8	96.4514	807	96.9534

**Table 6:** First column shows the channel label, the second column shows the initial training accuracy, and third one shows the marked correction by a neurologist and the last one shows the final accuracy.

Channel	Accuracy after initial training (%)	Number of epochs marked by the user	Accuracy after retraining (%)
Fp1	89.0861	57	89.7390
Fp2	89.0432	57	90.0443
F3	92.5991	57	92.3307
F4	89.6807	57	90.1430
C3	91.9454	57	90.8942
C4	91.1039	57	90.9973
P3	89.3490	57	90.3629
P4	89.7354	57	89.7726
O1	89.1013	57	90.2593
O2	89.2719	57	91.0779
F7	91.9906	57	92.4641
F8	91.7540	57	91.6937
T3	93.4416	57	93.9856
T4	93.2478	57	92.9901
T5	91.3400	57	92.1312
T6	92.8583	57	92.6363
Fz	88.8938	57	89.7298
Cz	84.0064	57	85.7228
Pz	91.6708	57	92.0561
E	85.2267	57	85.5279
PG1	84.6921	57	85.2892
PG2	85.3189	57	85.3886
A1	91.6580	57	92.0482
A2	90.4476	57	90.6291
T1	84.2211	57	85.0355
T2	87.7382	57	87.7849
X1	94.3820	57	94.7490
X2	94.0092	57	94.1500
X3	86.2930	57	86.6199
X4	84.5908	57	85.6526
X5	77.4855	57	79.1122
X6	88.1346	57	89.2410
X7	80.6365	57	81.5108

**Table 7:** First column shows the channel label, the second column shows the initial training accuracy, and third one shows the marked correction by a neurologist and the last one shows the final accuracy.

Channel	Accuracy after initial training (%)	Number of epochs marked by the user	Accuracy after retraining (%)
FP1F7	94.0520	807	94.7708
F7T7	94.7174	807	95.8794
T7P7	93.2686	807	94.7645
P7O1	95.6134	807	96.6610
FP1F3	93.6408	807	95.0283
F3C3	91.8523	807	93.3858
C3P3	91.9485	807	92.6572
P3O1	93.4837	807	94.4496
FP2F4	93.2756	807	94.1740
F4C4	91.0014	807	92.1137
C4P4	96.1309	807	96.4253
P4O2	88.8798	807	90.5184
FP2F8	91.0906	807	92.0630
F8T8	92.3974	807	93.8587
T8P8	94.3950	807	95.4360
P8O2	93.6889	807	93.7203
FZCZ	87.1507	807	87.6928
CZPZ	90.0607	807	91.9421
P7T7	93.7488	807	95.0216
T7FT9	93.1506	807	94.5268
FT9FT10	95.8059	807	96.3352
FT10T8	95.0270	807	95.8781

**Table 8:** Classification rate improvement caused by retraining. First column shows the channel label, the second column shows the initial training accuracy, and third one shows the marked correction by a neurologist and the last one shows the final accuracy.

Channel	Accuracy after initial training (%)	Number of epochs marked by the user	Accuracy after retraining (%)
Fp1	84.1736	57	84.6666
Fp2	85.9321	57	86.2142
F3	89.3863	57	90.1669
F4	82.5886	57	84.0065
C3	84.6526	57	85.6825
C4	84.0094	57	87.0688
P3	82.8481	57	84.6240
P4	83.0264	57	85.0625
O1	84.6445	57	84.1711
O2	83.3906	57	84.7017
F7	85.0919	57	88.3044
F8	84.8240	57	86.7992
T3	87.8007	57	88.8983
T4	92.0082	57	92.0256
T5	83.5051	57	86.4678
T6	85.5538	57	87.3744
FZ	83.2279	57	83.9403
CZ	80.0350	57	82.1546
PZ	90.5629	57	91.4475
E	87.3452	57	86.5264
PG1	88.4426	57	88.3773
PG2	83.0150	57	83.3391
A1	84.1149	57	85.5487
A2	84.0726	57	85.0169
T1	81.1019	57	83.0590
T2	83.5085	57	86.0844
X1	85.4925	57	87.7204
X2	85.2431	57	86.6259
X3	78.6937	57	80.5123
X4	81.2826	57	81.3851
X5	73.0822	57	77.2667
X6	83.9101	57	84.7386
X7	76.2795	57	79.2632
